# Preclinical In Vitro Model to Assess the Changes in Permeability and Cytotoxicity of Polarized Intestinal Epithelial Cells during Exposure Mimicking Oral or Intravenous Routes: An Example of Arsenite Exposure

**DOI:** 10.3390/ijms23094851

**Published:** 2022-04-27

**Authors:** Pravin Parajuli, Kuppan Gokulan, Sangeeta Khare

**Affiliations:** Division of Microbiology, National Center for Toxicological Research, US-Food and Drug Administration, Jefferson, AR 72079, USA; parajuli32@gmail.com (P.P.); kuppan.gokulan@fda.hhs.gov (K.G.)

**Keywords:** arsenic, xenobiotic, immunotoxicity, in vitro model, intestinal permeability, oral exposure, intravenous exposure, translational model

## Abstract

The gastrointestinal tract (GIT) is exposed to xenobiotics, including drugs, through both: local (oral) and systemic routes. Despite the advances in drug discovery and in vitro pre-clinical models, there is a lack of appropriate translational models to distinguish the impact of these routes of exposure. Changes in intestinal permeability has been observed in different gastrointestinal and systemic diseases. This study utilized one such xenobiotic, arsenic, to which more than 200 million people around the globe are exposed via their food, drinking water, work environment, soil, and air. The purpose of this study was to establish an in vitro model to mimic gastrointestinal tract exposure to xenobiotics via oral or intravenous routes. To achieve this, we compared the route (mimicking oral and intravenous exposure to GIT and the dose response (using threshold approach) of trivalent and pentavalent inorganic arsenic species on the permeability of in vitro cultured polarized T84 cells, an example of intestinal epithelial cells. Arsenic treatment to polarized T84 cells via the apical and basolateral compartment of the trans-well system reflected oral or intravenous routes of exposure in vivo, respectively. Sodium arsenite, sodium arsenate, dimethyl arsenic acid sodium salt (DMA^V^), and disodium methyl arsonate hydrate (MMA^V^) were assessed for their effects on intestinal permeability by measuring the change in trans-epithelial electrical resistance (TEER) of T-84 cells. Polarized T-84 cells exposed to 12.8 µM of sodium arsenite from the basolateral side showed a marked reduction in TEER. Cytotoxicity of sodium arsenite, as measured by release of lactate dehydrogenase (LDH), was increased when cells were exposed via the basolateral side. The mRNA expression of genes related to cell junctions in T-84 cells was analyzed after exposure with sodium arsenite for 72 h. Changes in TEER correlated with mRNA expression of focal-adhesion-, tight-junction- and gap-junction-related genes (upregulation of *Jam2, Itgb3* and *Notch4* genes and downregulation of *Cldn2, Cldn3, Gjb1,* and *Gjb2)*. Overall, exposure to sodium arsenite from the basolateral side was found to have a differential effect on monolayer permeability and on cell-junction-related genes as compared to apical exposure. Most importantly, this study established a preclinical human-relevant in vitro translational model to assess the changes in permeability and cytotoxicity during exposure, mimicking oral or intravenous routes.

## 1. Introduction

The human gastrointestinal tract (GIT) covers almost 400 m^2^ of area and helps in digestion and selective absorption of the food, water, and other nutrients we eat. The gastrointestinal tract is a long tube consisting of villi, microvilli, mucus membrane, secretory cells, immune cells and many other components. It helps in the absorption of food, creates a barrier to unwanted materials in the body and protects from the leakage of nutrients [1,2]. The GIT receives 10–15% of total blood pumped from the heart. The lumen of the GIT is exposed to xenobiotics via the oral route because of the food and water we consume. The GIT is also exposed to xenobiotics present in the blood through systemic circulation [3]. Since the GIT is connected to the outside and the inside of the body, appropriate function of the GIT and its barrier function are important for maintaining proper health. Impairment of the GIT could result in diseases such as inflammatory bowel disease, irritable bowel syndrome, celiac disease, and obesity. Changes in intestinal permeability are also related to chronic diseases such as Alzheimer’s disease, Parkinson’s disease, diabetes, depression, and autism spectrum disorders [4]. Having a translational model to access oral and systemic effects of xenobiotics could be beneficial for preclinical evaluation of xenobiotics [4,5]. In this study, we use a T-84-cell monolayer using trans-epithelial electrical resistance (TEER) as a model to measure the effect of a commonly found xenobiotic, arsenic (and its species), that interacts with the GIT via the oral as well as intravenous routes [6,7,8,9].

Arsenic is one of the most common chemical toxicants in the environment. It is generally found in water, soil, mines, industrial sources, and rocks. Human exposure to arsenic can be through water, food, agricultural products, industrial products, drugs containing arsenic, and marine animal products [10,11,12]. The United States Environmental Protection Agency (EPA) has set the maximum level of arsenic contamination in water to be 10 μg/L or 10 ppb [13]. However, studies show that about 2.1 million people in the USA, and more than 200 million people worldwide, drink water from private wells in which the level of arsenic is higher than 10 ppb [14,15]. Even a single oral exposure to arsenic is known to cause changes to gastrointestinal tract (GIT) homeostasis, as measured by changes in the intestinal microbiome and mRNA expression of gut-associated immune-response-related genes [16,17].

Ingestion of agricultural products such as rice, grains, fruits, vegetables, wine and meat, where irrigation water or pesticides contain arsenic, is another source for arsenic interaction with GIT. Consumption of marine organisms from arsenic-contaminated water is also responsible for arsenic exposure to humans [18]. Arsenic exposure through inhalation and skin absorption occurs via industrial sources, mining, contaminated soil, volcanic environment, coal fired power plants, pesticides, tobacco smoke, and dust. Arsenic has been also used in the treatment of diseases, including psoriasis, asthma, and cancer, via intravenous injection. This route of exposure to arsenic leads to systemic circulation and thus reaches the GIT via mesenteric vessels [11,19,20,21,22,23]. Moreover, intravenous exposure to the fetus can occur during prenatal development as a consequence of parental exposure [9,24].

Orally ingested arsenic can be metabolized and converted to different arsenic species via the GIT before reaching systemic circulation. In the GIT, arsenic can react with the food and acid in the stomach or become metabolized by microbes present in the gut, enzymes in the mucosal membrane, and enzymes present in epithelial cells of intestine [11,25]. However, during intravenous exposure, arsenic bypasses metabolism by gut enzymes and microbiota (first pass effect) [6,9,21].

More than 100 different forms of arsenic species have been identified in the environment [26]. The effect of arsenic has been linked with its chemical form and oxidation state. Inorganic forms of arsenic show high toxicity [27]. The US Department of Health and Human Services has classified arsenic and inorganic arsenic compounds as carcinogens to humans (http://ntp.niehs.nih.gov/ntp/roc/twelfth/roc12.pdf, accessed on 29 August 2021). Among the inorganic forms of arsenic, trivalent arsenic is known to have more of a toxicological effect than the pentavalent form of arsenic. Methylated species of arsenic such as MMA and DMA are major methylated species of arsenic [28]. Arsenic is converted into its methylated species in biological systems, such as metabolism inside human body, marine organisms, and plants. MMA and DMA can cause oxidative stress, generation of reactive oxygen species (ROS), oxidative DNA damage, and cytotoxicity [29,30,31]. Based on the bioavailability of each species of arsenic, arsenic reaches systemic circulation and shows a toxicological effect in different organs [25]. Inorganic arsenic has 80–90% bioavailability upon oral ingestion. Uptake of arsenic through the skin is less than 40% [32,33]. Human exposure to arsenic is related to different toxicological effects such as organ damage, diabetes, gastrointestinal tract distress, immune diseases, dermatological diseases, reproductive dysfunction, and neuronal and cardiovascular diseases [10,28,34].

**Figure 1:**
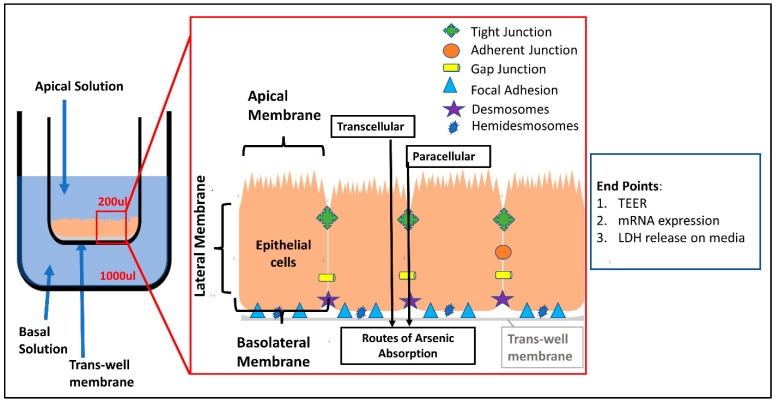
Experimental design and detail structure of the polarized monolayer of T-84 cells. Apical and basal exposures were conducted in individual wells. Apical exposure mimics the oral route, whereas basal exposure mimics the intravenous exposure. Expression of genes involved in the formation of tight junction, adherent junction, gap junction, focal adhesion, desmosomes and hemidesmosomes maintain the integrity of the epithelial cell monolayer.

Arsenic increases the free reactive oxidation species and causes oxidative stress and damage to the epithelial cells in the GIT [35]. The intestines have a large mucosal layer surface lined with epithelial cells, protecting the body from entry of toxic chemicals and harmful bacteria into the systemic circulation, while being selectively permeable to limited chemicals, water and nutrients. Transport of nutrients and other molecules from the GI lumen to systemic circulation is regulated by transcellular and intracellular/paracellular pathways [1,36]. Epithelial cells are attached to each other via the extracellular matrix by the presence of cell junction proteins (Figure 1). The cell junction proteins hold the cells tight to form a seal against the entry of xenobiotics by forming protein–protein interactions. Paracellular permeability of the intestines is determined by the expression level of these proteins. A change in expression of cell junction proteins can change intestinal permeability, which has been observed in different gastrointestinal and systemic disease conditions, including: inflammatory bowel disorder, infectious diarrhea, irritable bowel disorder, Crohn’s disease, and cancer [37]. Permeability changes can be observed in vitro by measuring the resistance of the monolayer of cells using trans-epithelial electrical resistance (TEER) [38], and by measuring the change in expression of genes regulating the cell junctions. Mainly, six different classes of cell junction proteins are present: tight junction, adherent junction, gap junction, focal adhesion, desmosomes and hemidesmosomes.

Previous studies have revealed that the intestinal flora help in conversion of arsenic to different species, and the effects are based on the extent of metabolism of arsenic [6,39]. Moreover, it has been shown that arsenic speciation is also dependent on the route of exposure of the arsenic (oral vs. other routes of exposure) since oral arsenic undergoes metabolism in GIT in the presence of gut enzymes, microbiota and interaction with food, whereas the majority of arsenic from other routes of exposure undergoes systemic biotransformation [12,31]. The exposure of a polarized intestinal epithelial cell model via the apical surface is representative of an exposure to xenobiotics via the oral route, as the cells grown in the trans-wells reflect the luminal area of the GIT with well-defined microvilli [40]. The exposure of xenobiotics from the basal chamber mimics the exposure via the mesenteric vessels or intravenous route, an area that has not been explored well until now. Although the EPA has set the maximum level of arsenic in water to be 10 μg/L, millions of people in the world are exposed to higher concentrations of arsenic. Ground water of some of the countries contains more than 1000 μg/L of arsenic [41]. The current study is designed to assess the effects of inorganic arsenic and its species (trivalent and pentavalent inorganic arsenic and methylated pentavalent arsenic species) using polarized intestinal epithelial cells that reflect both oral and IV exposure assessment through continuous monitoring of the changes in TEER up to 72 h post exposure. The concentrations of arsenic used for study were 12.8, 6.4, 3.2 and 1.6 µM, which represent the concentrations of arsenic above the concentration recommended by the EPA. These concentrations represented the threshold approach for dose response in our assay as suggested earlier [42]. This study could also help to understand the effect of arsenic present in the gastric lumen and in the blood. Furthermore, we assessed mRNA gene expression of permeability-related genes and cytotoxic effects as measured by the release of LDH.

## 2. Materials and Methods

### 2.1. Cell Line

Human T-84 colon carcinoma cells were purchased from ATCC and maintained in DMEM-F12 media supplemented with 5% FBS, penicillin/streptomycin and Amphotericin B. Media were changed every two days, and cells were monitored for any change in morphological structure. After the cells reached 80–90% confluency, subculture was performed as described earlier [40].

### 2.2. Chemicals

Sodium (meta) arsenite (CAS No. 7784-46-5) (trivalent), sodium arsenate dibasic heptahydrate (CAS No. 10048-95-0) (pentavalent), and dimethylarsinic acid sodium salt (DMA) (CAS No. 6131-99-3) (pentavalent) were purchased from Sigma (St. Louis, MO, USA). Disodium methyl arsonate hydrate (MMA) (CAS No. 144-21-8) (pentavalent) was purchased form Chem, Service Inc. (West Chester, PA, USA). Stock solution (10 mM) was freshly prepared in phosphate buffer saline and further diluted in cell culture media for the exposure studies. EGTA (ethylene glycol-bis (β-aminoethyl ether)-N,N,N′,N′-tetraacetic acid) was purchased from Sigma (St. Louis, MO, USA).

### 2.3. Treatment Concentration of Arsenic Species

T-84 cells were cultured in trans-well porous filter membrane until monolayer was formed. The wells that reached a monolayer, as monitored by the plateau stage in TEER, were only included in the study. In the trans-well, the apical portion held 200 µL of cell culture media and the basolateral portion held 1000 µL of cell culture media. The wells with monolayer of T-84 cells were divided into different groups: control, apical, basolateral, and EGTA. Then, 20 µL of media were removed from the well and replaced by 20 µL of media containing arsenic for the treatment. Next, 800, 400, 200 and 100 µM were prepared in cell culture media, and 20 µL of each was added to the wells to attain 12.8, 6.4, 3.2 and 1.6 µM of final concentration, respectively. For the apical group, arsenic was added to the apical media and for the basal group, arsenic was added to the basolateral media. For the control group, cell culture media were added, and for EGTA group, EGTA was added in cell culture media.

### 2.4. Culture of T-84 Cells and Trans-Epithelial Electrical Resistance (TEER) Study

T-84 cells were grown in T-75 (75 cm^2^) flasks for up to 80–90% confluency. Cells were trypsinized, centrifuged and resuspended in DMEM-F12 media, supplemented with 5% FBS, penicillin/streptomycin and Amphotericin B. Cells were seeded in a density of approximately 2 × 10^5^ cells/200 μL/well in the apical portion of a trans-well porous filter membrane. Cells were grown to monolayers as monitored by a plateau stage in TEER. Media were changed every 2–3 days. After cells reached a monolayer, cells were exposed with different concentrations of sodium arsenite, sodium arsenate, DMA and MMA for 72 h either from the apical or basolateral side. Treatment groups consisted of cell media only (control) and different concentrations of sodium arsenite, sodium arsenate, DMA and MMA (1.6, 3.2, 6.4 and 12.8 µM). EGTA (ethylene glycol-bis (β-aminoethyl ether)-N,N,N′,N′-tetraacetic acid) was used as positive control, as EGTA is known to chelate calcium and loosen the cell–cell adhesion. Cell media were used as treatment control. Treatments were performed two different sides (independent transwells), the apical side and basolateral side. The apical side mimics the luminal side of the intestine in the in vitro assay and the basolateral side mimics the interstitial side of the intestinal epithelium. After treatment, TEER was monitored for 72 h, and the changes in TEER were analyzed for the entire period. After 72 h, the apical solution, basal solution and the cellular monolayer were extracted and stored in different tubes. Apical and basal solutions were stored at −80 °C until used in lactate dehydrogenase assay for cellular toxicity assessment. Monolayers of cells were stored in the presence of TRIzol reagent (ThermoFisher, Waltham, MA, USA), used for extraction of mRNA for gene expression studies.

### 2.5. Analysis of Trans-Epithelial Electrical Resistance

To study the effects of arsenic and its species on the barrier function of intestine, we used the T-84 cell monolayer model and monitored the TEER function. The ECIS TEER24 (Applied Bioscience, Troy, NY, USA) was used to carry out the trans-epithelial electrical resistance to study the in vitro change in intestinal permeability. Resistance of the cell monolayer seeded in a 0.3 cm^2^ trans-well filter membrane was monitored in real time as a function of permeability. Data were collected as TEER (ohm·cm^2^) vs. time. The data for electrical resistance were converted to percentage change in TEER, and time of arsenic addition was considered as time “0”. Percentage change in TEER for every 1 h is reported in the graphs. All the treatments were performed in triplicate. The graphs represent the mean ± SD of percentage change in TEER. Assay was performed three times on independent days for each treatment and control groups.

Percentage change in TEER was calculated by using the following formula:

% change in TEER at time “t” = (TEER at time “t” − TEER at time “0”)/(TEER at time “0”) ∗ 100%

### 2.6. Lactate Dehydrogenase (LDH) Assay

Lactate Dehydrogenase Activity Assay Kit (Sigma, St. Louis, MO, USA) was used to determine the LDH release in T-84 cell culture media. Release of lactate dehydrogenase in the media is correlated with the disruption of the cell membrane and cell death. The method described in a report [43] was used for determination of the cytotoxic effect of sodium arsenate. After 72 h of treatment of T-84 cells monolayer in trans-well with sodium arsenate, the TEER experiment was terminated. After termination of the experiment, apical and basolateral cell media from each well were collected in separate Eppendorf tubes and stored at −80 °C until used. Apical and basolateral media were pooled and analyzed for the release of LDH in the media. The Triton X-100 surfactant was used as a positive control for cell lysis.

### 2.7. Extraction of mRNA, DNAse Treatment and cDNA Conversion T-84 Cells

The filter membranes along with the cells were collected after the treatment with arsenic species and stored in Eppendorf tubes containing TRIzol reagent. Chloroform was added, and the aqueous layer was transferred, precipitated and washed with 70% isopropanol. After washing and drying, the pellets were resuspended in nuclease free water. The extracted RNA was quantified using Cytation (BioTek, Winooski, VT, USA). The extracted mRNA was treated with DNAase kit (TURBO DNA-free Kit, Invitrogen, Carlsbad, CA, USA). The mRNA was converted to a complementary DNA strand by using SuperScript IV VILO Master Mix (Invitrogen, Carlsbad, CA, USA).

### 2.8. Determination of Cell-Junction-Related Gene Expression Using qPCR

RT² Profiler™ PCR Array Human Cell Junction Pathway Finder from Qiagen was used to study the focal adhesions, tight junctions, gap junctions, adherent junctions, desmosomes and hemidesmosomes genes. First, 9 ng of cDNA template was added along with the RT² SYBR Green ROX qPCR Mastermix in each well. The qPCR conditions were set as: 95 °C for 5 min, followed by 45 cycles of 10 s, denaturation at 95 °C, 10 s annealing at 55 °C, and 20 s elongation at 72 °C. The melting curve of all the samples was observed after running the experiment.

The baseline was adjusted for 16 cycles, and threshold cycle (Ct) values were reported. The data were analyzed by using Qiagen geneglobe analysis. All the apical and basal treatment groups were compared with the control group (cells without any treatment). More than a 2-fold change and *p* values less than 0.05 were considered to be significantly different than the control.

## 3. Statistical Analysis

All the assays were performed in triplicate, and the results were compared using paired *t*-test. Two-fold change (for gene analysis) and *p* values less than 0.05 were considered as a significant difference between the treatment and control group.

## 4. Results

### 4.1. Effect of Arsenic Species on the Intestinal Cell Monolayer Permeability

**Figure 2:**
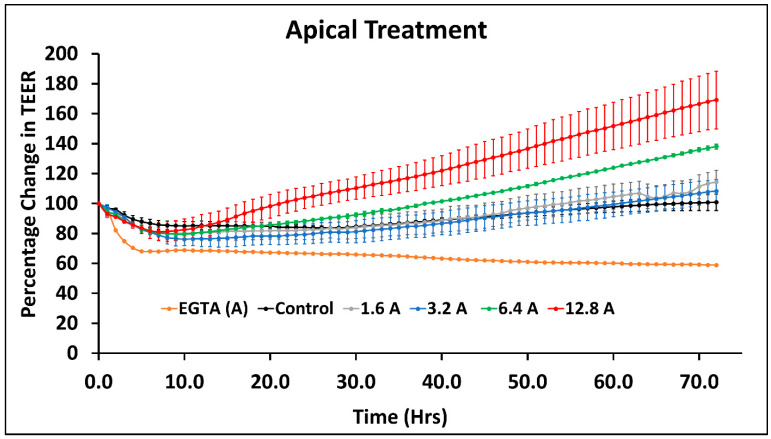
Change in TEER of T-84 cells monolayers during exposure to sodium arsenite from the apical side. A in legend denotes treatment from apical side with 1.6, 3.2, 6.4 and 12.8 µM concentrations. In addition, 2 mM of EGTA apical treatment was used as positive control. The graph represents the mean ± SD for each treatment group.

Treatment with sodium arsenite on the apical side caused an increase in resistance of the monolayer of cells; moreover, the higher concentration showed higher resistance. A change in TEER was not observed in 1.6 or 3.2 µM of sodium arsenite (Figure 2).

**Figure 3:**
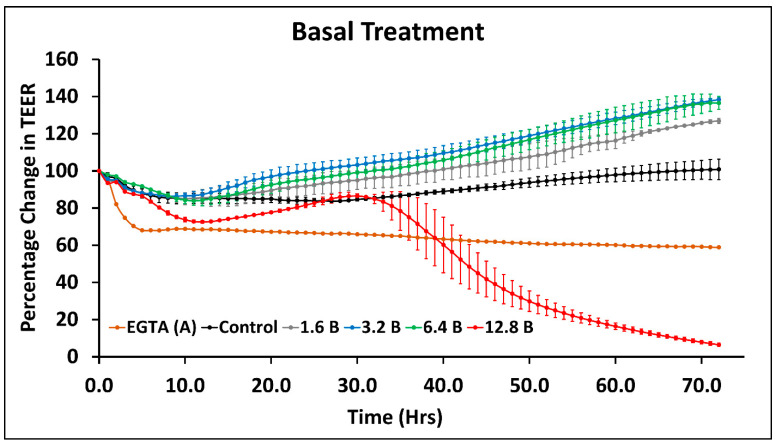
Change in TEER of T-84 cells monolayers during exposure to sodium arsenite from the basolateral side. B in legend denotes treatment from basolateral side with 1.6, 3.2, 6.4 and 12.8 µM concentrations. In addition, 2 mM of EGTA apical treatment was used as positive control. The graph represents the mean ± SD for each treatment group.

In the basolateral side of the treatment, up to a concentration of 6.4 µM, there was an increase in resistance (Figure 3). However, treatment with 12.8 µM of sodium arsenite on the basolateral side resulted in a decrease in resistance after 1 day of treatment (Figure 3).

As opposed to the treatment of sodium arsenite, treatment of T-84 cells with sodium arsenate (Supplementary Figure S1a,b), DMA (Supplementary Figure S2a,b) and MMA (Supplementary Figure S3) did not exhibit change in TEER. Overall, these TEER results show that the trivalent species of arsenic (sodium arsenite) changed the permeability of the T-84 cells monolayer, but treatment with pentavalent and pentavalent methylated species did not have any significant effect on the permeability of T-84 cells monolayer. Overall, among the tested arsenic species, the trivalent sodium arsenite was found to be more toxic to epithelial cell permeability when assessed by TEER, compared to pentavalent species and methylated species, DMA and MMA. As shown in Figure 1, the epithelial layer plays an important role as the first line of defense by maintaining the tight adhesion between the cells and attachment of the basolateral layer. Thus, we decided to determine the mechanism of toxicity of sodium arsenite by assessing the mRNA expression of genes of T-84 epithelial cells involved in the intestinal permeability study.

### 4.2. Effect of Sodium Arsenite on T-84 Cell-Junction-Related Genes When Exposed from Apical Side

An apical exposure model was used to mimic the exposure of arsenic toward the intestinal lumen. Arsenic is mostly found in drinking water and food. Apical exposure will mimic the arsenic present in food or water. To study the mechanism for different responses of T-84 cell monolayers in TEER studies, genes related to cell junction for the intestines were studied. Focal adhesions, tight junctions, gap junctions, adherent junctions, desmosomes and hemidesmosomes genes are the genes that play major roles in maintaining cell permeability and intestinal barriers. Changes in expression of the cell-junction-related genes might give us more mechanistic information regarding the change in permeability [44]. After 72 h of exposure of the T-84 cell monolayer to sodium arsenite, mRNA from each sample was harvested and converted to cDNA and the effect of exposure on cell-junction-related genes was studied. Focal adhesion, tight-junction- and gap-junction-related genes were significantly affected by the exposure of sodium arsenite (Table 1; Supplementary Figure S4).

The focal adhesion gene *Cav2* was downregulated by 3.2 and 1.6 µM of apical exposure, whereas *Itgb3* was upregulated by 6.4 µM of apical treatment. Tight-junction-related gene, *Cldn1*, was upregulated by 12.8 µM, and *Cldn15* was upregulated by 3.2 µM of sodium arsenite. Tight-junction-related genes *Cldn3, Cldn4, Esam, Ocln,* and *Tjp3* were downregulated by sodium arsenite treatment. Cldn3 was downregulated by 12.8, 6.4 and 3.2 µM of sodium arsenite. *Cldn4* was downregulated by 6.4 µM and *Ocln* was downregulated by 3.2 µM of sodium arsenite. *Esam* was downregulated by 12.8, 6.4, 3.2 and 1.6 µM of sodium arsenite. *Tjp3* was downregulated by 12.8, 6.4, and 1.6 µM of sodium arsenite. Gap-junction-related-gene *Gjb2* was downregulated by 12.8 µM of Sodium arsenite.

Overall, we could observe the downregulation of the genes *Cav2, Cldn3, Cldn4, Esam, Ocln, Tjp3 and Gjb2* after arsenic exposure, and the genes *Itgb3, Cldn1,* and *Cldn15* were upregulated. More than 10-fold regulation was observed in downregulation of *Cldn3* genes at 3.2 µM of sodium arsenite.

### 4.3. Effect of Sodium Arsenite Exposure on T-84 Monolayer and Change in Cell-Junction-Related Gene Expression

Exposure of the T-84 cells monolayer with sodium arsenite from the basolateral side was monitored for 72 h using the TEER function. Cells harvested after the endpoint of 72 h study were used for the assessment of change in expression of cell-junction-related genes, using the RT-PCR method. The mRNA expression results show that focal-adhesion-, tight-junction-, gap-junction- and adherent-junction-related genes were affected by the treatment of sodium arsenite (Table 1).

More of the tight-junction-related genes were affected by the exposure of sodium arsenite. Exposure of sodium arsenite could upregulate *Cldn1*, *Cldn6*, *F11r* and *Jam2* genes related to tight junction, whereas genes such as *Cldn2*, *Cldn3*, *Cldn4*, *Cldn7*, *Esam*, and *Tjp3* related to tight junction were downregulated. Sodium arsenite at 12.8 µM could upregulate *Cldn1, Cldn6, F11r* and *Jam2* genes, whereas it could downregulate genes such as *Cldn2*, *Cldn3*, *Cldn4*, *Cldn7*, *Esam*, and *Tjp3*. Sodium arsenite at 6.4 µM could upregulate *Cldn1* and *Jam2* genes and downregulate *Cldn3*, *Esam* and *Tjp3* genes. Sodium arsenite at 3.2 µM could upregulate the *Cldn1* gene and downregulate *Cldn3, Esam* and *Tjp3* genes, whereas 1.6 µM downregulated genes such as *Cldn3*, *Cldn4*, *Esam*, and *Tjp3*.

Regarding the focal-adhesion-related genes, exposure of sodium arsenite could upregulate the *Itgb3* gene, but downregulated *Cav1*, *Itga1*, and *Itgb2* genes. Exposure to 12.8 µM sodium arsenite could upregulate *Cav1*, *Itga1*, and *Itgb2* genes, 6.4 µM downregulate *Itga1* and *Itgb2* genes, and 3.2 µM downregulate Itgb2 gene. The *Itgb3* gene was upregulated by 12.8, 6.4, and 1.6 µM of sodium arsenite.

Gap-junction-related genes *Gjb1* and *Gjb2* were downregulated by exposure of T-84 cells to sodium arsenite. *Gjb1* was downregulated after exposure to 12.8, 6.4, 3.2 and 1.6 µM concentration, and *Gjb2* was downregulated by 12.8 and 6.4 µM of sodium arsenite. Less numbers of adherent junction genes were affected by sodium arsenite exposure. Sodium arsenite at 12.8 µM could downregulate the *Notch2* and upregulate *Notch4* gene.

Overall, the results show that *Cldn2*, *Cldn3*, *Cldn4*, *Cldn7*, *Esam*, *Tjp3 Cav1*, *Itga1*, *Itgb2*, *Gjb1*, *Gjb2* and *Notch2* were downregulated after exposure of T-84 cells to sodium arsenite and the genes *Cldn1*, *Cldn6*, *F11r*, *Jam2*, *Itgb3*, and *Notch4* were upregulated after exposure to sodium arsenite. More than 10-fold regulation was observed in downregulation of *Cldn2, Cldn3, Gjb1, and Gjb2* genes, whereas the same was observed in upregulation of *Jam2*, *Itgb3* and *Notch4* genes. Concentration-dependent effects were only observed in downregulation of *Gjb1, and Gjb2* genes.

These results indicate that exposure of arsenic from the basolateral side has a more adverse effect on the expression of the permeability-related genes. Change in permeability as observed in the TEER studies can be related to the change in expression of permeability-related genes. To determine if these changes can have any impact on cell toxicity, levels of lactate dehydrogenase (LDH) in the media were assessed.

### 4.4. Effect of Sodium Arsenite on Cell Cytotoxicity

**Figure 4:**
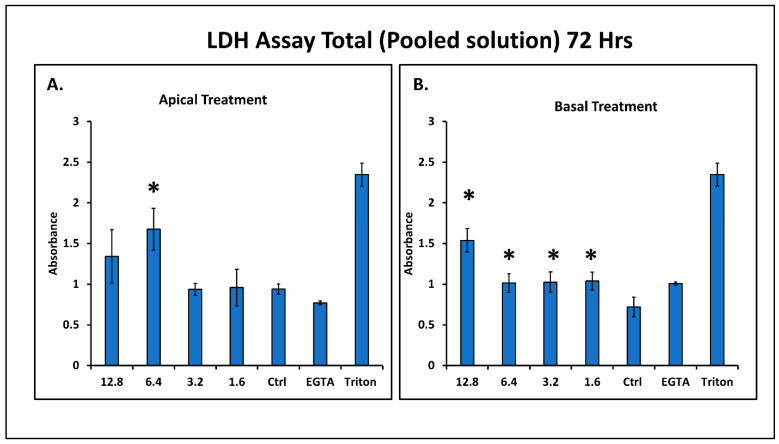
Effect of the exposure of sodium arsenite on release of LDH. LDH release was measured in the pooled cell culture supernatant (apical + basal), after apical (**A**) and basal (**B**) treatment with the sodium arsenite concentrations 1.6, 3.2, 6.4 and 12.8 µM or 2 mM of EGTA (as positive control). Triton was used as a positive control for cell lysis. Absorbance was measured at 450 nm at the endpoint. (* *p* < 0.05).

Pooled apical and basolateral media were analyzed for the release of LDH to study the mechanism of change in TEER with 12.8 µM basolateral treatment of sodium arsenite. LDH is known to be released in cell media after the lysis of cells [45]. Therefore, LDH was measured to determine the cytotoxicity of sodium arsenite. Treatment with sodium arsenite from the apical side showed a significant increase in LDH release for 6.4 µM only (Figure 4A). In contrast, the cytotoxic effect (LDH release) was observed in treatments with all concentrations of sodium arsenite (Figure 4B). Thus, from this observation, we can conclude that sodium arsenite is more cytotoxic when cells are exposed from the basolateral side of the monolayer of intestinal cells.

## 5. Discussion

This study provides a preclinical in vitro translational model to study the effects of xenobiotics in intestinal permeability and compares oral vs. systemic exposure. Toxicity of arsenic and its species have been studied for a long time. People exposed to higher concentrations of arsenic through food or water were reported to have problems related to skin, immune function, reproduction, gastrointestinal function, cardiovascular function, and neuronal functions [10]. Trivalent arsenic species is regarded as the more toxic form of arsenic as compared to other species [46]. This study was designed to observe the dose response (using a threshold approach) of different species of arsenic when they are treated from the apical vs. basolateral side of the intestines in an in vitro condition to depict oral or intravenous exposures to GIT [42]. Exposure of the same amount of sodium arsenite from the basolateral side caused different effects on the permeability of the monolayer of intestinal epithelial cells as compared to exposure from the apical side. The effect of sodium arsenite on intestinal permeability was more prominent when the arsenic was exposed from the basolateral side of the T-84 cell monolayer. Sodium arsenite also caused significant higher perturbation for the mRNA expression of cell-junction-related genes when the arsenic exposure was from the basolateral side, an exposure route that mimics exposure to GIT via mesenteric vessels or the intravenous route of exposure. Treatment from the apical side caused changes in fewer genes and a lower-fold regulation (when compared with basolateral exposure). These results are in line with the in vivo treatment of sodium arsenite when CD-1 mice were exposed via the oral or intravenous route (manuscript under preparation). This study also investigated the possible cause of difference for the effect of sodium arsenite exposure from the apical vs. basolateral side to that which mimics oral vs. absorbed arsenic (intravenous exposure) on intestinal permeability. When the T-84 cells monolayer was exposed to sodium arsenite for 72 h, a marked reduction in permeability was observed in the basolateral treatment group at 12.8 µM. The decrease in resistance might be due to loosening of the cell junction or due to cytotoxic effects of sodium arsenite from the basolateral side. The difference in effects with the same treatment on opposite sides also helps to validate the importance of this model of in vitro treatment. However, this effect might be due to polarization of cells or to secretion of mucin from cells in the apical compartment, which might work as a protective layer, a true reflection of xenobiotic exposure via the oral route, in vivo. Furthermore, the basolateral exposure model also mimics the possible route of exposure of absorbed xenobiotics during prenatal development as a consequence of parental exposure.

T-84 cells are epithelial cells derived from colorectal carcinoma. T-84 cells have been widely used for studying intestinal permeability, as they resemble the epithelial cells of the gastrointestinal lumen and they are less prone to differentiation [47]. It is also known that they resemble the villi-like structure in the in vitro culture medium after formation of the monolayer, and they secrete mucin in in vitro conditions. This makes the T-84 cell monolayer a suitable model for the study of intestinal permeability and cell junction pathway genes [48]. Furthermore, T-84 cells are appropriate for the study of effects using TEER, and they are a suitable system for studying the intestinal epithelium [49]. Epithelial cells in the intestines help in the absorption of food and nutrients as well as act as a protective barrier for entry of harmful chemicals and pathogens in the body [50]. Changes in permeability and integrity of the intestinal epithelial barrier could change the absorption pattern. Increases in permeability could also help the pathogenic chemicals and microbes to enter the blood stream, which can cause diseases [51]. During the cytotoxicity assay (as assessed by the LDH release), both EGTA and Triton were used as a positive control in LDH analysis. Since it is well established that Triton disrupts the cell membrane and causes LDH to release in the medium; Triton was used as positive control for the experiment. Our experiment shows here that the reduction in TEER due to EGTA was not because of the cytotoxic effect.

In this study, the expression of different permeability-related genes was studied to find the mechanism of differential effect of sodium arsenite exposure to the apical and basolateral sides of the T-84 cell monolayers. It was evident from the result that more of the change in expression of permeability-related genes was induced by the basolateral exposure of sodium arsenite as compared to the apical treatment. The effect could be correlated to a higher number of downregulated genes during basolateral exposure of sodium arsenite.

Epithelial cells are held together to form a tight barrier by tight junctions. Tight junctions lie toward the tip of the lateral side of two connecting epithelial cells. Tight junctions maintain the compartmentalization of body fluids by forming a tight connection between two epithelial cells. This helps to separate the apical fluid from the basolateral fluid. Tight junctions control the permeability of epithelial cells [52] and have a role in controlling diffusion across the paracellular pathway. Changes in the expression of tight junction proteins could impair intestinal permeability [53]. Impaired tight junctions have been found in several diseases such as ulcerative colitis, Crohn’s disease, celiac diseases, and metabolic disorders [37]. Tight junctions consist of a group of proteins that join the cell–cell connection with the actin cytoskeleton inside the cells. Occludins, tight junction proteins zonula occludens (TJP), claudins, junctional adhesion molecule (JAM), endothelial cell adhesion molecule (ESAM) and tricellulin are the prominent members of tight junction proteins [54]. Claudins help in paracellular channel function, and this family of protein has 27 transmembrane proteins. The properties and biological activity of all the members are not completely understood [55]. In the present study, increased expression has been observed in *Cldn1*, *Cldn6* and *Cldn15* genes, whereas downregulation is observed in the expression of *Cldn2*, *Cldn3 and Cldn4* genes after the cells are exposed to sodium arsenite. More than 20-fold downregulation of *Cldn2*, and *Cldn3* genes occurred in the basal treatment group. Reduction in *Cldn3* expression level has been linked with impaired intestinal barrier and inflammatory bowel disorder [56]. *Jam2* gene expression was increased after exposure with sodium arsenite. The *Jam2* protein helps in anchoring the cells during normal conditions and during cell migration. The *Jam2* gene was found to be upregulated in gastric adenocarcinoma tissues [57].

Focal adhesion connects the extracellular matrix to the cell cytoskeleton and connects the intestinal epithelial cells to the basement of the intestines. It consists of the Caveolin and Integrin families of proteins [58]. In this study, we observed that the cells exposed to sodium arsenite increased expression of the *Itgb3* genes. Exposure of cells with 12.8 µM of sodium arsenite from the basolateral medium resulted in increased expression by 36.9-fold. *Itgb3* is regarded as a marker for the migratory behavior of cells. Some of the studies have correlated the expression of *Itgb3* with the progression of epithelial-to-mesenchymal transition (EMT) and invasiveness of the cells [59]. An increase in the permeability from TEER studies with the exposure of sodium arsenite could be explained as the migratory property of cells.

Gap junctions are the small channels between two cells that help in cell–cell communication and in the exchange of signals, metabolic factors, growth signals, and cell differentiation signal. It allows for communication between the adjacent cells through small hemichannels. The role of the gap junction is important for proper communication of neurons, heart, and smooth muscles such as the intestines [60]. Gap junctions also help in intestinal motility and in the conductance of signals [61]. In this study, the expression of gap junction-related genes *Gjb1* and *Gjb2* are found to be markedly reduced after the cell monolayer is exposed to sodium arsenite. In one study, deficiency of the Connexin32 (encoded by *Gjb1* gene) was found to induce tumor formation in the lungs and liver [62]. An increase in Connexin 26 (encoded by *Gjb2* gene) has also been found to be related to apoptosis of tumor cells (encoded by *Gjb1* gene). An increase in Connexin 26 was found to inhibit tumorigenesis of cells [63]. A reduction in expression of *Gjb1* and *Gjb2* after exposure to sodium arsenite could have inhibited apoptosis and induced epithelial to mesenchymal transformation.

Adherens junctions play role in cell–cell connection and help cells to attach to each other. Adherens junctions consist of Cadherins, Delta, Nectin, and Notch receptors [64]. In this study, exposure to sodium arsenite (12.8 µM) from the basolateral side increased the expression of the *Notch4* gene. An increase in *Notch4* expression has been correlated with migratory, invasive, and tumor-like properties of cells [65]. Upregulation in the *Notch4* gene could also be a protective response from the cells in an effort to maintain homeostasis of the intestinal T-84 cells monolayer in the culture.

**Figure 5:**
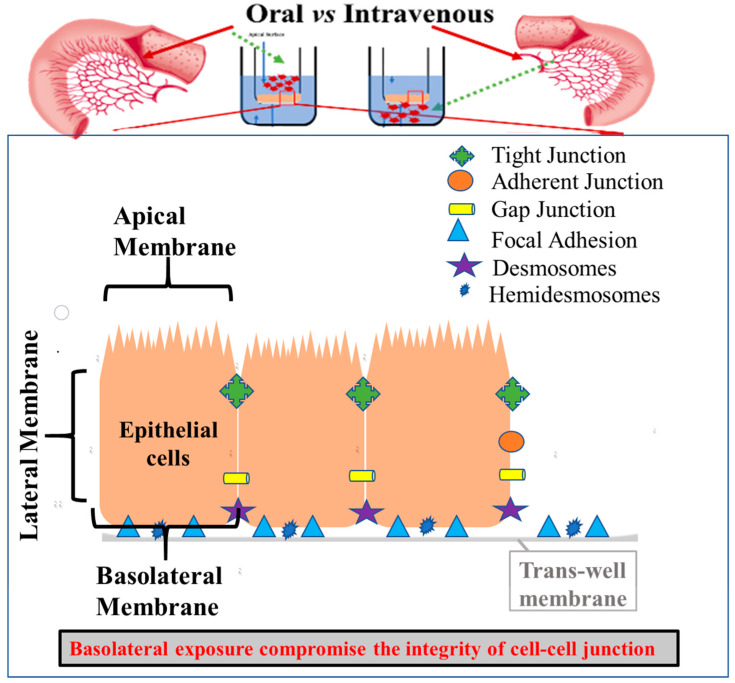
Schematic diagram depicts the detailed structure and exposure routes of the polarized T-84 cells cultured in the trans-well. Exposure of polarized epithelial cells from the apical side mimics oral exposure, whereas exposure from the basal side mimics intravenous exposure. Epithelial cells are attached to each other and with the extracellular matrix by the presence of cell junction proteins. The cell junction proteins hold the cells tight to form a seal from entry of xenobiotics, by forming protein–protein interaction. Paracellular permeability of the intestines is determined by the expression level of these genes. A higher number and larger magnitude of the change in expression of cell junction genes can change intestinal permeability. Here, basolateral exposure resulted in compromised cell–cell junction integrity.

Thus, we can summarize that the effects of sodium arsenite are more prominent when the T-84 cell monolayer is exposed to arsenic from the basolateral side (mimics the intravenous route of exposure) as compared to the apical side (mimics oral exposure). Effects of sodium arsenite were not significant with a lower dose but were prominent at a concentration of 12.8 µM. The permeability change was related to changes in focal adhesion, tight junctions, and gap junctions as shown in Figure 5. Furthermore, this study also utilized an in vitro model to mimic GIT exposure of various xenobiotics via oral or intravenous routes. This system could be also used to evaluate the potential fate and impact of xenobiotics on the intestines of a fetus during prenatal development as a consequence of parental exposure.

## Figures and Tables

**Table 1:** Fold regulation of permeability-related genes in T-84 cells after exposure to different concentrations of sodium arsenite from apical or basolateral compartment. Here, numbers represent the fold regulations compared to control group, more than 2-fold regulations are bolded, and statistically significant change compared to control group are colored. Green color represents upregulation, and red color represents downregulation.

Focal Adhesions						Focal Adhesions					
	Apical 12.8	Apical 6.4	Apical 3.2	Apical 1.6	Apical EGTA		Basal 12.8	Basal 6.4	Basal 3.2	Basal 1.6	Basal EGTA
**CAV1**	**−5.16**	−1.99	1.09	−1.55	−1.33	**CAV1**	**−3.77**	−1.53	−1.07	1.17	−1.35
**CAV2**	−1.86	−1.16	**−5.81**	**−3.52**	−1.37	**CAV2**	1.71	1.57	**2.73**	**2.91**	1.41
**ITGA1**	**−5.09**	−1.22	−1.60	1.28	**3.41**	**ITGA1**	**−4.50**	**−7.54**	1.75	**−3.28**	1.69
**ITGB2**	−1.37	1.09	1.98	1.39	**5.36**	**ITGB2**	**−5.42**	**−5.84**	**−3.59**	−1.85	1.02
**ITGB3**	**6.89**	**5.43**	1.40	1.01	**−2.83**	**ITGB3**	**36.90**	**9.43**	**6.20**	**11.40**	1.24
**Tight Junctions**						**Tight Junctions**					
	**Apical 12.8**	**Apical 6.4**	**Apical 3.2**	**Apical 1.6**	**Apical EGTA**		**Basal 12.8**	**Basal 6.4**	**Basal 3.2**	**Basal 1.6**	**Basal EGTA**
**CLDN1**	**7.12**	**7.11**	**2.56**	**2.96**	1.58	**CLDN1**	**5.17**	**4.37**	**5.22**	**3.74**	1.11
**CLDN15**	**3.96**	**4.00**	**8.61**	**3.32**	**5.88**	**CLDN15**	**3.54**	**2.30**	1.60	1.38	1.14
**CLDN2**	**−3.29**	**−3.44**	−1.74	1.01	**2.06**	**CLDN2**	**−37.65**	**−2.86**	−1.69	−1.03	−1.12
**CLDN3**	**−5.46**	**−8.63**	**−19.88**	**−2.31**	1.39	**CLDN3**	**−26.09**	**−16.75**	**−36.27**	**−7.14**	1.25
**CLDN4**	−1.82	**−2.22**	−1.08	−1.17	1.44	**CLDN4**	**−7.12**	**−2.31**	**−2.48**	**−3.40**	−1.61
**CLDN6**	**2.51**	1.10	**−6.53**	1.15	1.37	**CLDN6**	**7.78**	1.05	1.87	**2.03**	1.00
**CLDN7**	−1.54	−1.74	−1.08	1.04	1.62	**CLDN7**	**−4.25**	−1.81	−1.48	**−2.25**	−1.34
**ESAM**	**−7.28**	**−4.11**	**−6.34**	**−3.08**	1.02	**ESAM**	**−2.66**	**−5.22**	**−2.84**	**−5.07**	−1.05
**F11R**	1.26	−1.99	1.41	1.57	1.84	**F11R**	**2.71**	−1.03	−1.24	−1.41	1.07
**ICAM1**	**2.01**	**2.85**	**5.14**	1.78	**2.32**	**ICAM1**	**−2.90**	1.49	1.81	**2.12**	−1.64
**JAM2**	**3.24**	**2.58**	−1.14	−1.12	1.14	**JAM2**	**14.71**	**10.71**	**13.35**	**9.49**	**3.39**
**OCLN**	−1.00	**−3.37**	**−3.05**	−1.21	1.40	**OCLN**	1.14	−1.08	−1.71	−1.72	1.33
**TJP2**	1.49	1.10	−1.05	1.53	**2.50**	**TJP2**	−1.54	−1.07	1.12	−1.05	1.44
**TJP3**	**−6.95**	**−3.89**	−1.58	**−2.53**	−1.08	**TJP3**	**−8.48**	**−3.61**	**−2.67**	**−2.41**	−1.35
**Gap Junctions**						**Gap Junctions**					
	**Apical 12.8**	**Apical 6.4**	**Apical 3.2**	**Apical 1.6**	**Apical EGTA**		**Basal 12.8**	**Basal 6.4**	**Basal 3.2**	**Basal 1.6**	**Basal EGTA**
**GJB1**	**−11.35**	**−3.07**	−1.58	−1.05	1.69	**GJB1**	**−82.02**	**−20.71**	**−10.19**	**−7.14**	−1.35
**GJB2**	**−2.87**	**−2.51**	−1.29	−1.18	−1.45	**GJB2**	**−12.99**	**−3.78**	**−2.10**	**−3.98**	−1.64
**Adherent Junctions**						**Adherent Junctions**					
	**Apical 12.8**	**Apical 6.4**	**Apical 3.2**	**Apical 1.6**	**Apical EGTA**		**Basal 12.8**	**Basal 6.4**	**Basal 3.2**	**Basal 1.6**	**Basal EGTA**
**NOTCH2**	**−4.73**	−1.76	−1.16	**−3.25**	−1.15	**NOTCH2**	**−6.25**	1.02	**2.35**	1.15	−1.66
**NOTCH4**	**2.44**	**2.60**	1.04	**2.76**	**3.85**	**NOTCH4**	**13.22**	**3.34**	**2.42**	**2.37**	**2.26**

Red: downregulated; Green: upregulated.

## Data Availability

Data will be available on request.

## Supplementary Materials

The following supporting information can be downloaded at: https://www.mdpi.com/article/10.3390/ijms23094851/s1.
