# Supplementary material for: Preclinical In Vitro Model to Assess the Changes in Permeability and Cytotoxicity of Polarized Intestinal Epithelial Cells during Exposure Mimicking Oral or Intravenous Routes: An Example of Arsenite Exposure
Source: Int J Mol Sci. 2022 Apr 27;23(9):4851. doi: 10.3390/ijms23094851 (PMC9101442; doi:10.3390/ijms23094851)
Supplement: Supplementary file 1 [file ijms-23-04851-s001.zip › ijms-1707413-supplementary.pdf]

Supplemental Material

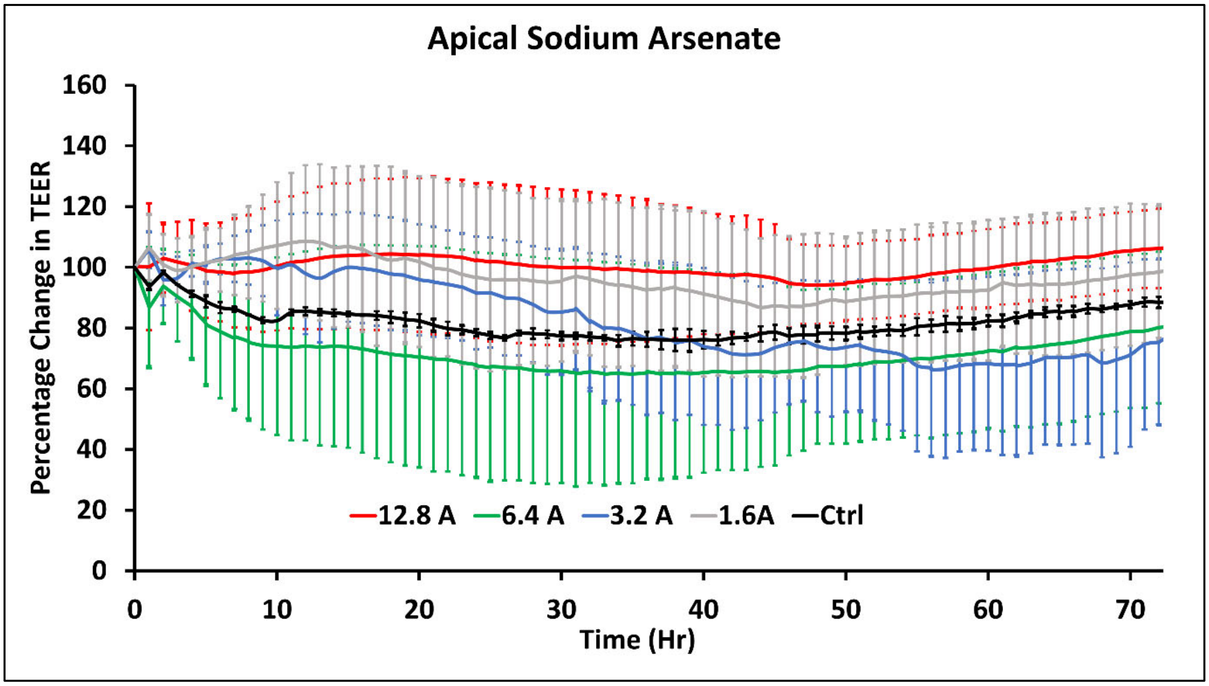

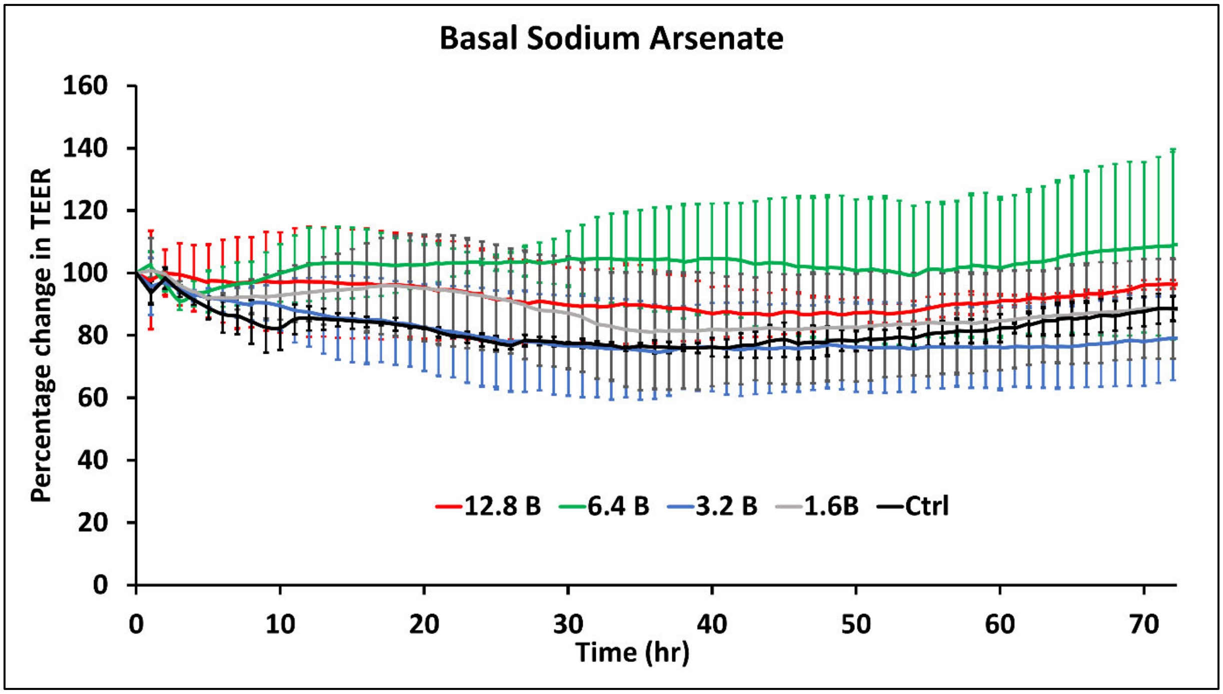

Supplemental Figure S1a (top) and S1b (bottom): Change in TEER of T-84 cells monolayers during the exposure of sodium arsenate from the apical side (Top panel; 1a). Change in TEER of T-84 cells monolayers during the exposure of sodium arsenate from the basolateral side (Top panel; 1b)

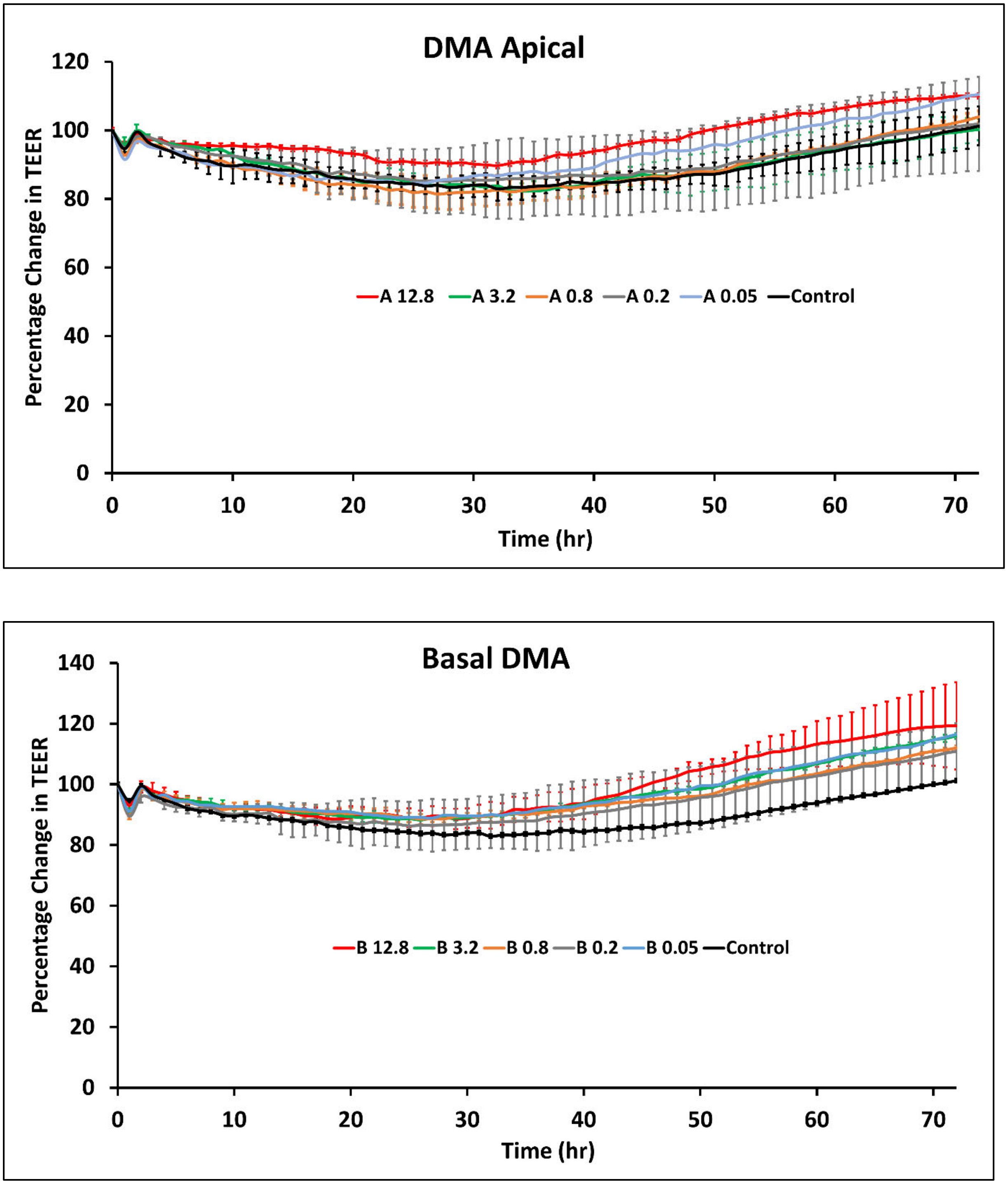

Supplemental Figure S2a (top) and S2b (bottom): Change in TEER of T-84 cells monolayers during the exposure to DMA from the apical side (Top panel; 2a). Change in TEER of T-84 cells monolayers during exposure of the DMA from the basolateral side (Lower panel; 2b)

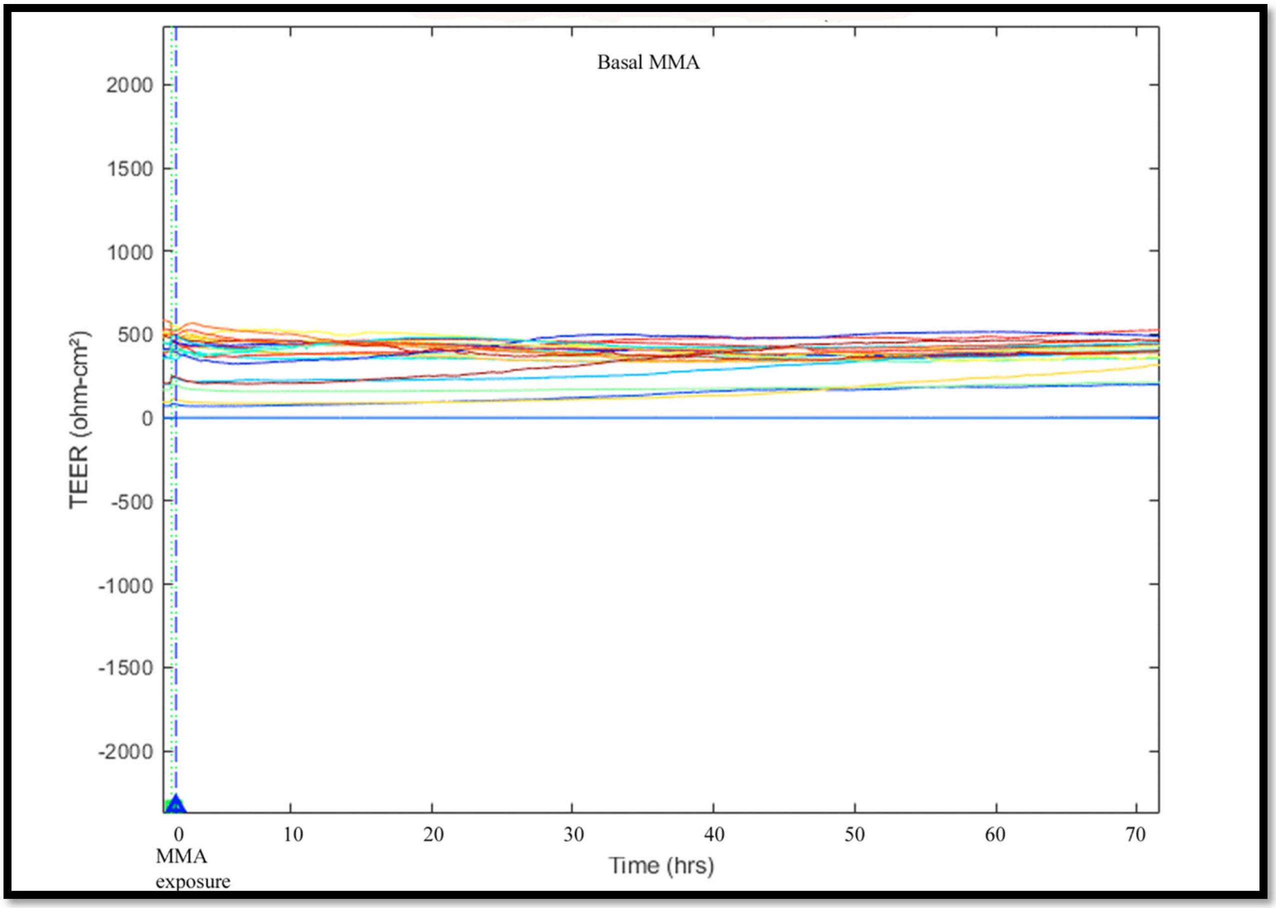

Supplemental Figure S3: MMA exposure from the basolateral side did not show any changes in the TEER of T-84 cells monolayers

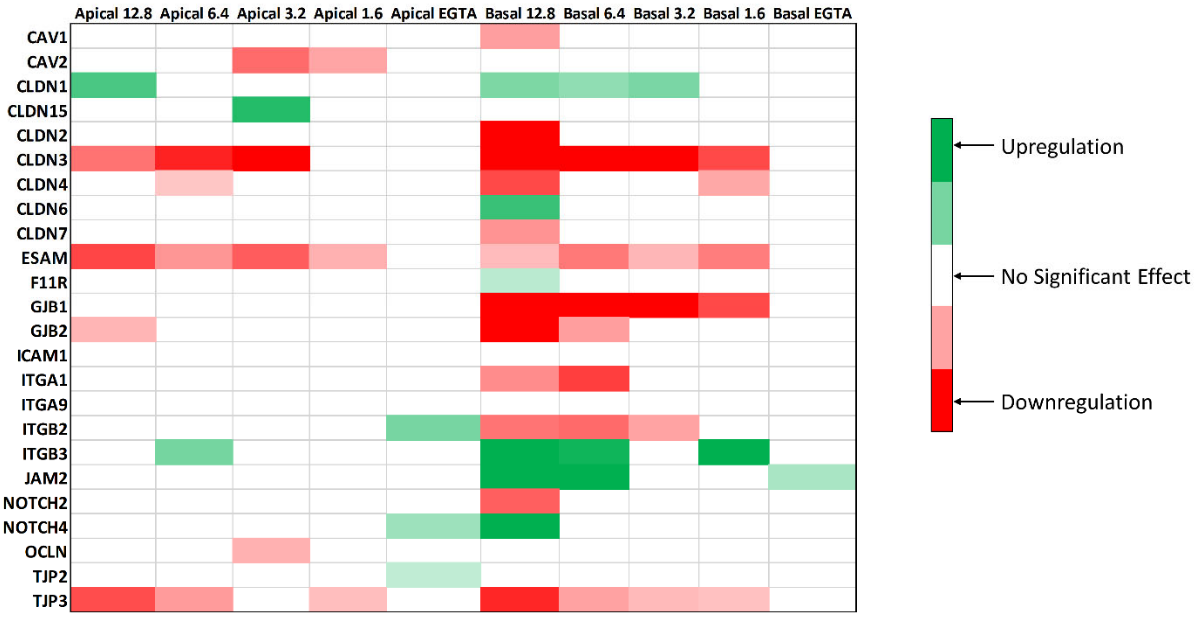

Supplemental Figure S4: Heatmap of the fold regulation of permeability related genes in T-84 cells after exposure to different concentration of sodium arsenite from apical or basolateral compartment. Green color represents the upregulation, red color represents downregulation.
